# Integrity of the Oral Tissues in Patients with Solid-Organ Transplants

**DOI:** 10.1155/2012/603769

**Published:** 2012-01-29

**Authors:** Gonzalo Rojas, Loreto Bravo, Karina Cordero, Luis Sepúlveda, Leticia Elgueta, Juan Carlos Díaz, Blanca Urzúa, Irene Morales

**Affiliations:** ^1^Department of Oral Pathology, Faculty of Odontology, University of Chile, Sergio Livingstone 943, Independencia, 8390-492 Santiago, Chile; ^2^Dental and Maxillo Facial Service, Clinical Hospital of University of Chile, Santos Dumont 999, Independencia, 8380456 Santiago, Chile; ^3^Faculty of Odontology, University of Valparaíso, Carvallo 211, 2360004 Valparaíso, Chile; ^4^Cardio-Vascular Department, Clinical Hospital of University of Chile, Santos Dumont 999, Independencia, 8380456 Santiago, Chile; ^5^Medicine Department, Clinical Hospital of University of Chile, Santos Dumont 999, Independencia, 8380456 Santiago, Chile; ^6^Department of Community and Basic Sciences, Faculty of Odontology, University of Chile, Sergio Livingstone 943, Independencia, 8390-492 Santiago, Chile

## Abstract

The relationship between the use of immunosuppressants in solid-organ transplant patients and oral tissue abnormalities has been recognized. The objective of this study was to determine the state of oral tissue integrity in renal, heart, and liver transplant patients who are on continuous medical and dental control. Forty patients of both sexes were clinically evaluated at the Clinical Hospital of the University of Chile to identify pathologies of oral mucosa, gingival enlargement (GE), decayed, missing, filled teeth (DMFT) index, and salivary flow. The average age of the transplant subjects was 49.4 years, and the age range was 19 to 69 years. Most subjects maintained a good level of oral hygiene, and the rate mean of DMFT was 14.7. The degree of involvement of the oral mucosa and GE was low (10%). Unlike other studies, the frequency of oral mucosal diseases and GE was low despite the fact that these patients were immunosuppressed. Care and continuous monitoring seem to be of vital importance in maintaining the oral health of transplant patients.

## 1. Introduction

Organ transplantation is a widely used treatment for the functional failure of an organ. The life expectancies of patients who have received heart, lung, kidney, liver, or bone marrow transplants have improved substantially in recent years [1], partly due to improvements in surgical techniques and the immunosuppressive drug therapies used to prevent transplant rejection [1]. From 1992 to 2009 in Chile, there have been a total of 4570 solid-organ transplants, including 3494 kidney transplants, 768 liver transplants, and 198 heart transplants [2].

Immunosuppressive therapy has a number of short- and long-term effects including infection, increased cardiovascular risk, and neoplasm that may threaten the patient's life [3, 4]. This treatment depresses the cellular immune response [5]. Cyclosporin A, the most widely used immunosuppressive drug, acts selectively on T-cell-mediated immune responses [1]. Clinically, this effect means a high risk of oral infections and associated complications. In patients who are treated with immunosuppressants, oral pathogens can cause local destruction and opportunistic infections due to the inability of the immune system to suppress and destroy pathogens. Lesions in the oral cavity could develop as a result of these side effects or drug interactions [5].

At the periodontal level, gingival enlargement (GE) associated with immunosuppressive therapy with cyclosporin A usually appears within the first 12 months of use [6], and the risk is increased with the concomitant use of calcium channel blockers and poor oral hygiene [7–10]. Studies have reported the presence of GE in 7–74.1% of renal transplant patients [11–15], and 22% of liver transplant patients are reportedly affected [4]. In a study that evaluated patients with heart, liver, and kidney transplants, 43% presented GE, and the major influencing factor was the blood concentration of cyclosporin A, followed by plaque and gingivitis levels [10].

Studies of renal transplant patients show a low percentage of lesions in the oral mucosa, with the most common lesions being candidiasis [11–17], herpes simplex infections [15, 17], hairy leukoplakia [13–15], fissured tongue [12], and lip cancer [15]. In liver transplant patients, fissured tongue is the most prevalent oral mucosa injury [4], although oral ulcers [18] and xerostomia [4] have also been observed. In heart transplant patients, increased frequencies of candidiasis [16], herpes simplex virus infection [19], and oral ulcers [20, 21] were observed in the oral cavities of patients.

The purpose of this study was to evaluate the status of oral tissues in patients who received heart, liver, and kidney transplants at the Clinical Hospital of the University of Chile José Joaquín Aguirre between 2008 and 2009 by determining the history of caries, the level of oral hygiene, and the presence of GE and oral mucosal lesions. Salivary flow and xerostomia status were also assessed.

## 2. Materials and Methods

A descriptive cross-sectional study was conducted from 2008 to 2009 with a sample of 22 males and 18 females (40 patients) who had received solid-organ transplants at the Hospital of the University of Chile, including heart (8 patients), liver (17 patients), and renal (14 patients) transplants and one combined heart and kidney transplant. The study was approved by the institutional Ethics Committee, and all patients expressed their willingness to participate through an informed consent form. We included patients who were 18 years of age or older, were transplant recipients, and had undergone transplantation at least 6 months prior to the study. Exclusion criteria included patients who refused to participate in the study, patients with reduced or altered cognitive function, and patients whose transplants were not functional and required new transplants.

 Data from each patient were collected following a set protocol, which included demographic data, general medical history, oral and dental history, intraoral clinical examination, and record of salivary flow and condition of xerostomia. In the general medical history, the type of transplant, the time elapsed since transplantation, and current medications were recorded. For the oral and dental history, patients were asked whether they had received pretransplant dental evaluations or used prosthetic devices. Patients were also asked about their oral hygiene habits, including the frequency of brushing and the use of mouthwash and dental floss.

 All patients were examined in a dental chair under direct light at the Dento Maxillo Facial Department of the hospital. The instruments used were a no. 5 mouth mirror, gloves, and a mask, and a photographic record was taken for each patient. Clinical examination was performed by an operator who was previously trained for each of the evaluated variables and a required kappa value greater than 0.6. The history of cavities was defined through the DMFT index, which was measured by visual inspection. At the periodontal level, the level of oral hygiene was assessed through the Simplified Oral Hygiene Index of Greene and Vermillion [22], and GE was defined as an overgrowth of the gum with a lobed granular appearance that covered at least one-third of the buccal crown. Both assessments were made by visual inspection. At the level of the oral mucosa, we assessed the presence of oral mucosal lesions associated with solid-organ transplantation [1]: recurrent labial herpes simplex, oral candidiasis, oral ulcers, hairy leukoplakia, fissured tongue, and lichenoid reaction. Finally, salivary flow was measured through the oral salivary flow rate test of Schirmer [23].

 For all variables, we performed descriptive analyses through tables, graphs, and numerical representations of measures of central tendency and dispersion using Stata 11.0 for Windows. The chi-square test was used to compare nominal variables between groups of transplant patients. The Kruskal-Wallis test was used for ordinal variables, and ANOVA was used for quantitative variables. For all tests, a value of *P* < 0.05 was considered significant, with a confidence interval of 95%.

## 3. Results

The sample consisted of 40 people with an average age of 49.4 years and an age range of 19 to 69 years. The age difference between men and women was not significantly different. The average age was  42 ± 16.3  years for heart transplant patients,  40 ± 11.4  years for renal transplant patients, and 58.2 ± 7.7 years for liver transplant patients.

 Most of the patients received single-organ transplants. The elapsed time since transplantation was highly variable and ranged from 6 to 192 months (16 years), with an average time of 57.8 months. Renal transplant patients had the longest period since transplantation, with an average of 84.2 months. No relationship was found between patient age and time since transplantation (Table 1).

 One 67-year-old patient received a heart-kidney double transplant, with a 51-month survival for both transplants. Five patients were excluded because their time since transplantation was less than 6 months. Women had lower average times since transplantation than men. 

Most of the patients took between three and five drugs, mainly immunosuppressants, and cyclosporin and corticosteroids were the most common drugs. Another type of commonly used drug was antihypertensives (Table 2). There was no difference between men and women in the number of drugs taken. 

 Most patients reported a tooth brushing frequency of two to three times daily. However, a significant proportion of women reported a frequency of four times per day, which was significantly higher than the frequency reported by the men. This high reported frequency of brushing is consistent with the oral hygiene index, which was low for most patients. Thus, 85% of patients had an index score lower than 1. Notably, 16 patients had a score of 0, indicating a very low level of bacterial plaque. 

The history of damage from caries (DMFT) showed an average of 14.7 teeth affected by caries, either from carious cavitation or loss by decay. There was no gender difference in the level of damage from caries. 

**Figure 1:**
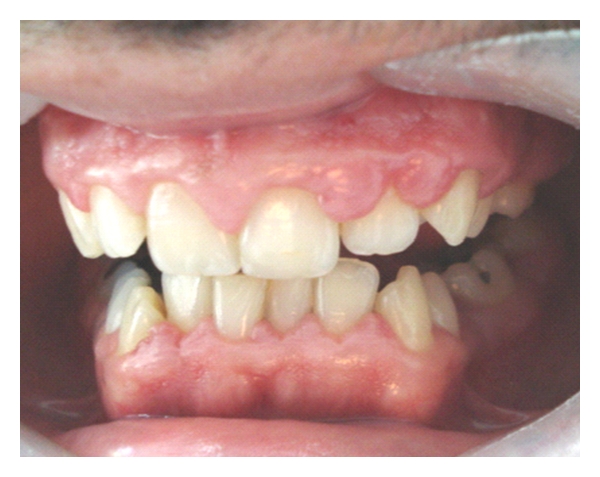
Gingival enlargement can be seen covering part of the coronal portion along with the absence of plaque or calculus (25-year-old male heart transplant patient who had cyclosporin treatment only).

**Figure 2:**
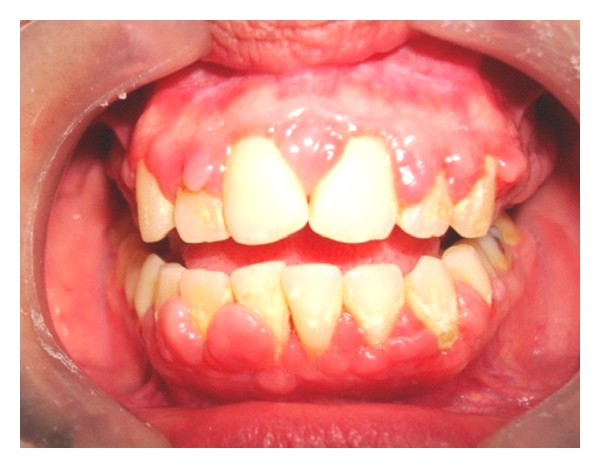
Gingival enlargement can be seen with signs of inflammation in a patient with plaque and calculus (31-year-old female renal transplant patient who was treated with cyclosporin, mycophenolate, and corticosteroids).

**Figure 3:**
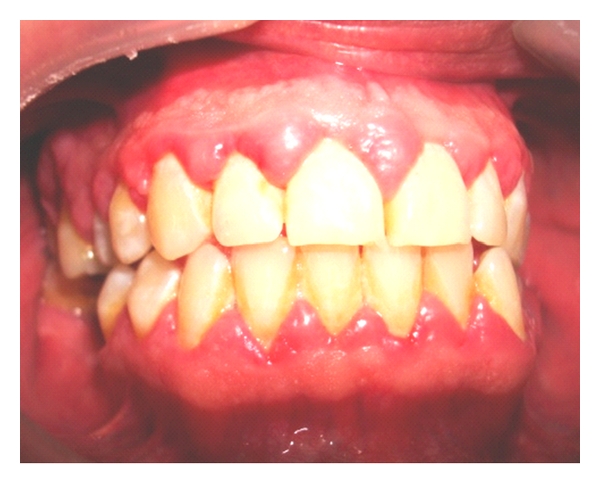
Gingival enlargement with signs of inflammation and presence of dental calculus in the lower third of the dental crowns (49-year-old male kidney transplant patient who was treated with cyclosporin).

Only four patients had GE, three of whom were men. Most patients exhibited good periodontal protection (Figures 1, 2, and 3). 

 Oral mucosal lesions were rare. There was no difference in the frequency between males and females for each oral lesion (Table 1). The majority of oral mucosal lesions involved fissured tongue, a relatively common condition in the general population. Candidiasis was observed in three male patients. No cases of hairy leukoplakia, a condition usually described in immunosuppressed patients, were observed. 

A descriptive analysis was conducted for oral mucosal lesions associated with immunosuppression and its relation to the time elapsed since transplantation. On one hand, in the three cases of candidiasis, the time of transplantation was under five years (mean 35,3 months). On the other hand, AG cases presented a wide time range elapsed since transplantation (from 16 to 120 months), and no relation was observed between these variables. The same analysis was conducted for drugs intake and oral lesions. The results showed no relation between development of oral pathology with type and number of immunosuppressive medications. 

Salivary flow was preserved for the majority of patients. Only three patients became hyposalivatory.

## 4. Discussion 

 This study aimed to assess the state of oral tissues in patients who received heart, liver, and kidney transplants at the Hospital of the University of Chile José Joaquin Aguirre between 2008 and 2009 by determining the history of caries, the level of oral hygiene, and the presence of GE and oral mucosal lesions. 

 Patients in the sample were adults, with no age differences between men and women. At the time of the study, kidney transplant patients had the lowest average age, and liver transplant patients had the highest average age. This age range is similar to the ranges reported in previous studies, which also show that renal transplant patients generally have a lower average age compared to liver and cardiac transplant patients (Table 3).

 The results of this study show that the frequency of oral mucosal pathologies was lower than that expected for this type of patient. The immunosuppressed condition is associated with the presence of several diseases, including candidiasis, hairy leukoplakia, and herpes virus infections, which are promoted by the reduced expression of cellular and humoral effector mechanisms. The low occurrence in this study may be explained by methodological reasons related to sample selection. Transplantation must have occurred at least 6 months prior for the patient to be included in the study. If the inclusion criteria had required a minimum of 2 years since receiving the transplant, then perhaps a larger number of oral mucosal lesions would have been observed, mainly due to noninfectious diseases. However, the time variable should not be relevant to the discovery of infectious diseases. 

 Alternatively, it can be assumed that the rigorous and close clinical monitoring of patients promotes general health conditions that tends to keep opportunistic infections commonly associated with immunosuppression under control. Others variables like nutritional status [24], hygiene habits [25], denture wearers, hyposialia, or the psychological status [26] can modulate the expression of infectious pathology in immunosuppressed patients. For example, some studies show that patients who receive hematopoietic stem cell transplantation have greater difficulty with oral hygiene habits than no transplanted patients [27], which is probably due to their xerostomic condition. 

Maybe oral hygiene habits are the most important to prevent oral infectious diseases in immunosuppressed patients. Goldman suggests that all transplantation patients and their caregivers should be educated regarding the importance of maintaining good oral and dental hygiene to reduce the risk for oral and dental infections. The maintenance of safe oral hygiene after transplantation can minimize the severity of infections and facilitate healing of mucositis. Consultation with physicians or transplant coordinators, frequent recall and oral prophylaxis, and daily antibacterial mouth rinses, all indicating dental care, consideration of antibiotic prophylaxis for invasive procedures, and careful screening for head and neck cancers, are suggested for dental treatment and care of the stable posttransplant patient [25]. We think that in our study the patients are being taken care of under these recommendations; therefore, they have good oral tissues conditions and a lower oral diseases frequency.

A close clinical monitoring can reduce the immunosuppression effect on the development of infectious disease of oral mucosal through the healthy behavior promotion like good feeding, physical exercise, oral hygiene, or better psychological attitude toward health care. 

Candidiasis is an opportunistic infection that is common in immunosuppressed patients and is more common in transplant patients than that in nontransplant patients [16]. Several studies have reported an average rate of candidiasis higher than 15% for renal transplant patients. In our study, a lower percentage of patients (7.5%) had clinical candidiasis infections. This result agrees with the results obtained by Dongari-Bagtzoglou et al. [16] in a study of renal and cardiac transplant patients. This low occurrence of candidiasis is not explained by the use of antifungal treatment because only one patient received this treatment. However, we must keep in mind that candidiasis do not only depends on systemic condition. Local factors are also important mainly the salivary flow. Our patients do not have a significant hyposialia, which is a risk factor for the fungal infection [28]. Therefore, this finding could explain the lower percentage of candidiasis than that in other studies. Regrettably, these do not inform about salivary, flow and comparisons are not possible. 

In our study, two cases of candidiasis were found in liver transplant patients, and one case was found in a patient who had a double cardiac-renal transplant. The ages of these three patients were above 65 years, which is well above the average age for the study sample. Patient age could explain the fact that, in a context of noninfectious compromise of the oral cavity, three patients had this infection. Advanced age is a described risk factor for developing candidiasis [29], and, therefore, these cases might be explained by this risk factor. The relationship between the consumption of cyclosporin and candidiasis is known; however, two of the reported cases were not being treated with cyclosporine, but with other immunosuppressive drugs: tacrolimus in one case and mycophenolate in the other, so the risk is the same because of the higher dosages of mycophenolate and tacrolimus correlated with symptomatic mucosal candidiasis [30]. 

 GE has been described as a pathology produced by the consumption of some drugs, including phenytoin sodium and cyclosporin [31]. Our data show that GE was not a significant finding in our study, as only four cases were recorded. Other studies show higher frequencies of GE; percentages exceeding 70% of renal transplant patients have been reported [13]. The literature has reported that GE depends on the effects of the drugs used and that the level of bacterial plaque also plays a significant role in the hyperplasia suffered by periodontal tissues. Our findings support this observation because subjects who were enrolled in the study showed good oral hygiene with low plaque indexes. 

 Other diseases of the oral mucosa that are usually observed in transplant patients are hairy leukoplakia and herpes virus infections. In our study, no case of hairy leukoplakia was recorded, and there was only one case of recurrent labial herpes. Other studies have shown frequencies in the range of 3–12% for leukoplakia and 2–7% for herpes infection. It is likely that the smaller size of our sample was a factor in the very low frequency of these conditions. However, our findings are consistent with other studies in that both of these pathological conditions are less prevalent than candidiasis and GE in our sample. 

 Of the other oral mucosal diseases, only fissured tongue was observed frequently. Although several studies did not report data regarding the presence of fissured tongue, those that did report data showed a percentage exceeding 35% of the sample, which is higher than the frequency observed in the present study. Fissured tongue is a benign disorder of little clinical relevance and is not uncommon in a clinically healthy population. Darwazeh and Almelaih [32] showed a prevalence of 11.5% in normal subjects in Jordanian population. Koay et al. [33] found a similar result in dental outpatients in Malaysia (13.8%), and higher frequency than 25% has been described in denture wearers older patients. Therefore, it is not possible to make a direct association between the prevalence of this condition and the condition of a renal, heart, or liver transplant recipient. The difference in prevalence with the other studies may be related to ethnicity, the criteria used to diagnose fissured tongue, or the hyposialia condition. Several reports show the relationship between hyposialia and fissured tongue [34]. Maybe the lowest frequency of fissured tongue would be explained by the salivary flow maintenance in our patients. 

 Hyposalivation is not a common complication in transplant patients. In total, 0.6% of the subjects studied by López-Pintor et al. [17] presented with hyposalivation. A similar percentage was observed in our study. However, these observed frequencies are similar to those of a healthy population. This finding suggests that salivary flow is unaffected despite the many drugs that transplant patients receive. In our sample, a significant percentage of patients (90%) received antihypertensives, which are associated with hyposalivation; however, this symptom was not common in our study. 

 Most of the sample population took more than three drugs concomitantly. In total, 90% were under an immunosuppression regimen with cyclosporin and antihypertensives. A substantial fraction took oral corticosteroids. However, oral tissue damage was minimal despite the many drugs that were taken. We believe that this result is due to the close clinical supervision by professional teams in the respective transplant units of the hospital. 

 While acknowledging the impact of courtesy bias or social desirability phenomena when it comes to answering behavioral questionnaires, the results of this study show significant adherence to oral hygiene care. As shown in Table 1, almost 100% of the patients brushed their teeth two to four times per day. This finding is consistent with clinical findings of the oral hygiene index, which showed a vast majority of patients with a score below 1, meaning that the dental calculus or plaque covered less than a third of the coronal portion of the teeth. 

 The integrity of oral tissues for patients who participated in this research is good and clearly better than that observed in the general population of Chile. Oral health indicators show severe damage as a result of caries and periodontal disease in Chilean people [35]. Thus, the doctor-patient relationship is thought to be essential for these patients. The respective medical teams maintain close, permanent care relationships and are committed to all health aspects of their transplant patients. Additionally, the Clinical Hospital of the University of Chile has a Dento Maxillo Facial Department with highly trained professionals to care for transplant candidates, in which a strict clinical protocol is followed including instructions for maintaining good oral hygiene. In the cases presented in this study, all patients had attended a pretransplant evaluation. 

 The findings of this study suggest that despite the vulnerability to the development of oral diseases involved in immunosuppression in solid-organ transplant patients, these patients can exhibit appropriate maintenance of oral tissue integrity, provided they are properly assessed, treated, and trained for the prevention of complications arising from the use of drugs that are known for their deleterious effects on the oral mucosa and periodontal and dental structures.

## Figures and Tables

**Table 1:** Clinical and demographic characteristics of the study sample.

	Total	Men	Women	*P*
	40	22	18	
Age (years)	49.4 ± 13.9	50.5 ± 12.8	48.1 ± 15.3	
Type of transplant				
Liver	17	8	9	NS
Renal	14	10	4	NS
Cardiac	8	3	5	NS
Cardiac-renal	1	1	0	
Time since transplant (months)	57.8 ± 49	67 ± 57.6	46.4 ± 35.9	
Number of drugs consumed				
0–2	2	2	0	NS
3–5	37	19	18	NS
6 or more	1	1	0	NS

Total	40	22	18	NS

Frequency of brushing				
0-1 times per day	1	1	0	NS
2-3 times per day	31	20	11	NS
4 times per day	8	1	7	*P* < 0.01
Oral Hygiene Index				
0	16	10	6	NS
1	18	8	10	NS
2	6	4	2	NS
3	0	0	0	
Damage from caries (DMFT)	14.7 ± 6.7	15.4 ± 6.4	14.1 ± 7.1	NS
Gingival enlargement	4	3	1	NS
Oral mucosa lesions				
Fissured tongue	7	4	3	NS
Candidiasis	3	3	0	NS
Herpes simplex	1	0	1	NS
Canker sores	1	1	0	NS
Other	11	7	4	NS
No lesion	21	10	11	NS
Hyposalivation	3	1	2	NS

**Table 2:** Main drugs consumed.

Drugs	N°	%
Antihypertensives	32	80
Cyclosporin	31	77,5
Corticosteroids	23	57,5
Mycophenolate	16	40
Insulin	7	17,5
Tacrolimus	7	17,5

**Table 3:** Comparison of results with other studies.

Author	Type of transplant	No. of patients	Age (years)	Gingival enlargement (%)	Candidiasis (%)	Fissured tongue (%)	Coated tongue (%)	Hairy leukoplakia (%)	Labial herpes (%)
de la Rosa et al. [14]	R	90	31.4 ± 29	48.9	18.7	NR	22.2	12.2	7.8
Al-Mohaya et al. [13]	R	58	39.2 ± 12.8	74.1	15.50	NR	22.4	8.6	NR
López-Pintor et al. [17]	R	500	53.6 ± 13.4	NR	7.4	NR	NR	0.2	2.6
Diaz-Ortiz et al. [14]	L	53	57.6 ± 9.1	20.8	NR	39.6	28.3	3.8	3.8
Güleç and Haberal [12]	R	100	35.0 ± 11.0	39	26.0	35.0	22.0	NR	0.0
Sahebjamee et al. [11]	R	100	42.9 ± 12.4	7	16.0	NR	NR	NR	
Dongari-Bagtzoglou et al. [16]	R C	81 9	52.7 ± 11.9 56.4 ± 12.3	NR	7.8	NR	NR	NR	NR
Present study	R-C-L	40	49.4 ± 13.9	10	7.5	17.5	NR	0.0	2.5
	R	14	40 ± 11.4						
	C	8	42 ± 16.3						
	L	17	58.2 ± 7.7						

Type of transplant: R: renal, L: liver, C: cardiac.

NR: not reported.
